# 5-HT_FAsTR: a versatile, label-free, high-throughput, fluorescence-based microplate assay to quantify serotonin transport and release

**DOI:** 10.1038/s41598-024-56712-z

**Published:** 2024-03-19

**Authors:** Lina Bukowski, Markus Emanuel Strøm, Jens Lindengren Andersen, Jannick Bang Maesen, Lin Tian, Steffen Sinning

**Affiliations:** 1 Department of Forensic Medicine, Aarhus University, Palle Juul-Jensens Boulevard 99, 8200 Aarhus N, Denmark; 2 Department of Biochemistry and Molecular Medicine, University of California, Davis, CA 95616-8635 USA; 3 Max Planck Florida Institute for Neuroscience, Jupiter, FL 33458 USA

**Keywords:** Serotonin, Transporter, Depression, Biosensor, Organic cation transporter, Drug discovery, Pharmacology, Psychiatric disorders, ADHD, Depression, Post-traumatic stress disorder, Neuroscience, Cellular neuroscience, Diseases of the nervous system, Molecular neuroscience, Transporters in the nervous system

## Abstract

The neurotransmitter serotonin plays a pivotal role in mood and depression. It also acts as a vasoconstrictor within blood vessels and is the main neurotransmitter in the gastrointestinal system. In neurotransmission, released serotonin is taken up by serotonin transporters, which are principal targets of antidepressants and the psychostimulant, ecstasy. The investigation of serotonin transporters have relied almost exclusively on the use of radiolabeled serotonin in heterogenous end-point assays. Here we adapt the genetically encoded fluorescent biosensor, iSeroSnFR, to establish and validate the Serotonin (5-HT) Fluorescence Assay for Transport and Release (5-HT_FAsTR) for functional and pharmacological studies of serotonin transport and release. We demonstrate the applicability of the method for the study of a neuronal, high-affinity, low-capacity serotonin transporter (SERT) as well as an extraneuronal low-affinity, high-capacity organic cation transporter and mutants thereof. 5HT_FAsTR offers an accessible, versatile and reliable semi-homogenous assay format that only relies on a fluorescence plate reader for repeated, real-time measurements of serotonin influx and efflux. 5HT_FAsTR accelerates and democratizes functional characterization and pharmacological studies of serotonin transporters and genetic variants thereof in disease states such as depression, anxiety and ADHD.

## Introduction

The serotonergic system originates in the raphe nuclei and projects to almost all regions of the brain. Serotonin (5-hydroxytryptamine, 5-HT) is a modulatory neurotransmitter system in the brain involved in regulating sleep, appetite, cognition and memory and is an important mood regulator. Due to its function as mood regulator, 5-HT plays a key role in depression pathogenesis. After its release from vesicles into the synaptic cleft and potential binding to serotonin receptors, 5-HT is removed by the neuronal serotonin transporter (SERT), the extrasynaptic organic cation transporters (OCT) or the plasma membrane monoamine transporter (PMAT)^1,2^, thereby terminating the signaling by 5-HT. Reuptake of 5-HT into the neuron by SERT allows for the repackaging of the neurotransmitter into vesicles where it can be reused in subsequent serotonergic signaling events. Thus, the high-affinity, low-capacity monoamine transporter SERT belonging to the neurotransmitter:sodium symporters (solute carrier 6 family, SLC6) plays an important role in regulating neurotransmitter homeostasis in the brain^3^. Together with the low-affinity, high-capacity transporters OCT and PMAT, they play a major role in regulating monoamine (MA) signaling^4,5^, making them principal targets for medications against several psychiatric disorders such as Attention-Deficit/Hyperactivity Disorder (ADHD), anxiety and major depression^6,7^ as well as targets for psychoactive recreational drugs. Most antidepressants target the serotonergic system by stimulating serotonergic signaling. Inhibitors of SERT do so by blocking the transporter, thereby increasing synaptic concentrations of 5-HT. Whereas the classical tricyclic antidepressants (TCAs), such as imipramine (Fig. 4A), are not very selective for the serotonin transporter, newer antidepressants, such as S-Citalopram, fluoxetine and paroxetine (Fig. 4A), are Selective Serotonin Reuptake Inhibitors (SSRIs) by predominantly blocking SERT. The psychostimulant effect of many amphetamine-based designer drugs relies on the efflux of neurotransmitters^8,9^. How the reverse operation of neurotransmitter:sodium symporters may be induced is still a matter of debate^10,11^.

The gold standard to study the effect of drugs on monoamine transporters (MATs) are radiotracer uptake and inhibition assays^9,12^. Radiotracer uptake assays require tedious washing steps for the termination of the assay and as such, they are end-point assays, which again requires multiple parallel experiments for normalization and control conditions. Radiotracer assays may pose a health hazard to researchers and require special permits and training as well as dedicated waste disposal, facilities and equipment, making it prohibitive to many research groups. Although using radiolabeled substrates is sensitive and fairly straight forward for studying uptake and uptake inhibition of neurotransmitters via MATs, it is very limited in its capacity for studying neurotransmitter release by for example amphetamine-like compounds. Hence, radiotracer uptake assays are not capable of differentiating MAT inhibitors from MA releasers. To be able to differentiate amphetamine-like substrates from inhibitors in vitro, dynamic superfusion assays in both transfected cells and synaptosomes, static batch release assays and electrophysiology have been applied^9,12–16^, with each method presenting its own set of limitations. Even though superfusion assays have the major advantage of eliminating reuptake of already released substrate back into the cell, they consume a large amount of buffer and substances. Furthermore, the method has very low throughput and it is very time consuming to determine the half-maximal effective concentration (EC_50_) for release by superfusion. Static batch release assays show the advantage of very little substance consumption. Whereas radiotracer uptake assays and static batch release assays do not directly allow for continuous measurements over time, superfusion assays do have a temporal resolution. The requirement for expensive isotope-labeled substrates, special equipment and laboratory facilities as well as necessary training makes it attractive to develop more accessible alternatives with comparable performance and added benefits in handling and temporal resolution.

In the past two decades, the optical measurement of fluorescence became the favored method to replace radiotracers. The first optical tracer of monoamine neurotransmitters were fluorescent false neurotransmitters (FFNs). Sulzer and Sames combined the characteristic element of monoamine neurotransmitters, an aminoethyl group, with a coumarin system to engineer FFN511 and thus incorporated a fluorophore into the structure of the neurotransmitter^17^. FFN511 was only the first member of an expanding family of FFNs used to study synaptic transmission^18–20^. However, the structure of FFNs only vaguely resemble the endogenous substrate of monoamine transporters and thus it will always be questionable whether results obtained with FFNs directly translates to the uptake mechanism and molecular pharmacology of the cognate substrate. Other fluorescent substrates that can be used to study neurotransmitter uptake are 4-(4-(dimethylamino)styryl)-N-methylpyridinium (ASP+)^21,22^ and 4-(4-dimethylamino)-phenyl-1-methylpyridinium (APP+)^23^, fluorescent analogs of the neurotoxin 1-methyl-4-phenyl-4-propionoxypiperidine (MPP+). The development of genetically encoded indicators that report the presence of neurotransmitters by changes in fluorescence (as reviewed in^24–26^), provided a useful radiotracer-free tool to study changes in extracellular neurotransmitter levels. An assay that facilitates intracellular fluorescent indicators for glucose and glutamate (iGluSnFR^27^) is the oscillating stimulus transporter assay (OSTA)^28^ that allows to study substrate uptake (influx) and release (efflux), albeit with very low throughput, in a setup using a perfusion system and a fluorescence microscope. Recently, Herenbrink et al. generated cell lines with inducible expression of seven different genetically encoded fluorescent dopamine (DA) sensors to record DA release from cultured neurons as well as striatal brain slices^29^. They furthermore show, that a cell line expressing the extracellular GPCR-based DA sensor GRAB_DA2_ can be used to measure DA uptake and release via the human dopamine transporter (DAT). The described assay can be conducted using commonly available equipment like a plate reader or microscope and appears to be a radiotracer-free alternative with high throughput potential. However, membrane embedded biosensors are sensitive to changes in the plasma membrane or membrane potential and many therapeutics treating psychiatric disorders are acting at the monoamine receptors. Thus, GPCR-based biosensors may be intrinsically confounded in reporting the action of psychotropics. Furthermore, measuring a change in free, extracellular neurotransmitter concentrations as a consequence of transport is at best only an indirect measure of intracellular neurotransmitter concentrations. The ability of low-capacity transporters like SERT or DAT to significantly alter extracellular neurotransmitter levels in the usual conditions for in vitro transporter assays is highly questionable. Instead, an intracellular localization of the fluorescent biosensor is preferable when studying MA influx via MATs. Unger et al. developed the genetically encoded fluorescent 5-HT indicator iSeroSnFR that is based on a soluble periplasmic binding protein (PBP) and we showed that it can be expressed both extracellularly tethered to the membrane or in the cytoplasm as a soluble protein^30^.

We here present the 5-HT Fluorescence Assay for Transport and Release (5-HT_FAsTR), a fluorescence-based method that is radiotracer-free and suitable for high throughput assays. It reliably detects changes in label-free, intracellular 5-HT concentrations in response to 5-HT transport by both low-capacity, high-affinity serotonin transporters (SERT) as well as high-capacity, low-affinity serotonin transporters (OCTs). The versatility and high dynamic range of the 5-HT_FAsTR method enables the determination of key functional parameters of different serotonin transporters despite their highly different apparent substrate affinities and turn-over rates. Furthermore, 5-HT_FAsTR can differentiate 5-HT releasers from SERT inhibitors and produces high quality data with a high temporal resolution. We demonstrate how 5-HT_FAsTR can be used both as a general tool for functional characterization of transporter variants and as a screening tool for pharmacological characterization of serotonin transporter drugs capable of distinguishing between transporter inhibitors (influx blockers) and 5-HT releasers (efflux inducers) and we show how it can be used to determine their potencies. 5-HT_FAsTR enables accelerated drug discovery and makes the study of serotonin transporters accessible to a much wider number of laboratories and researchers.

## Results

### Development of a fluorescent biosensor based assay

**Figure 1:**
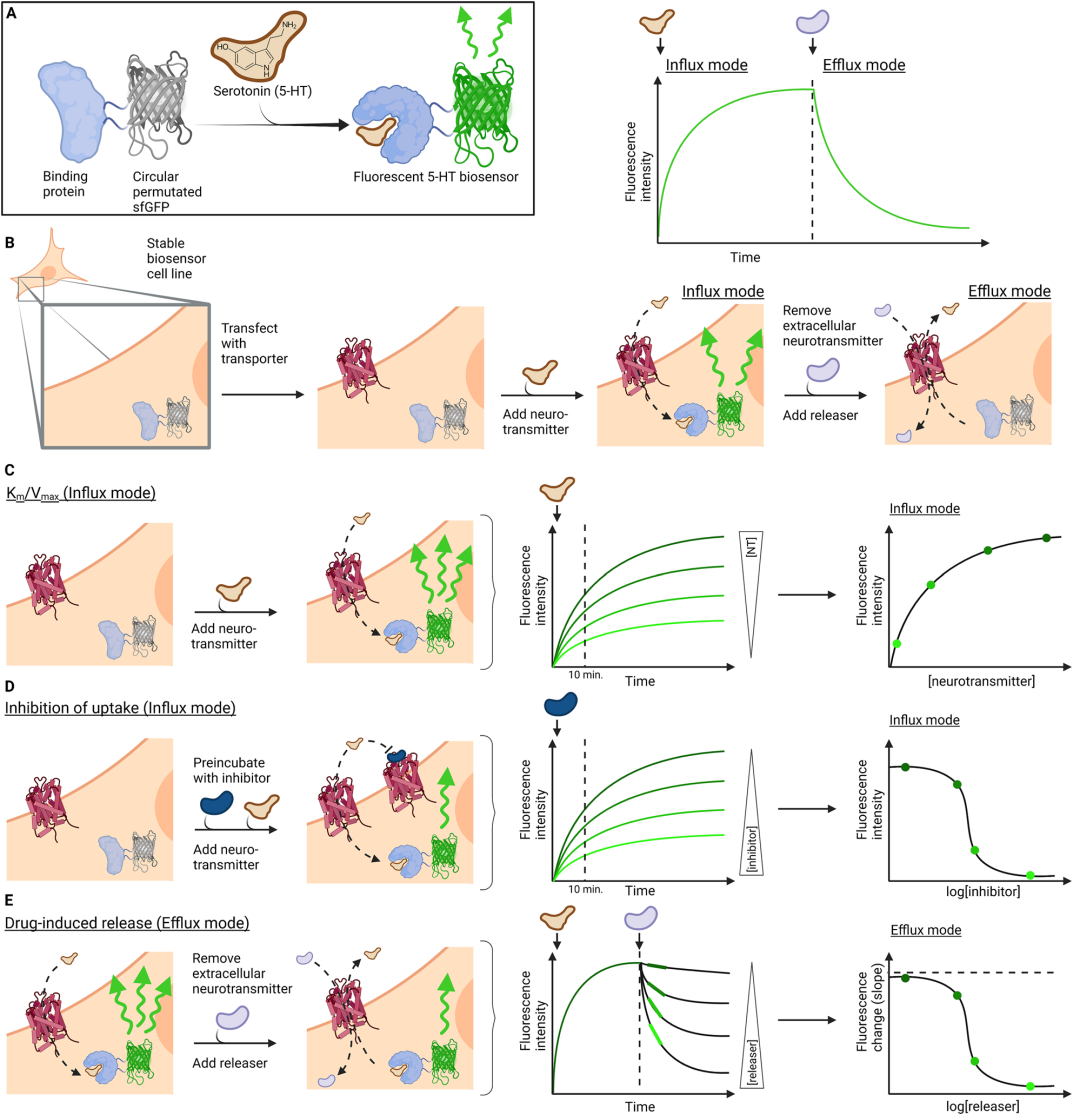
Scheme for 5-HT_FAsTR. (**A**) The biosensor iSeroSnFR consists of a periplasmic binding protein (PBP) able to bind 5-HT and a circular permutated super folder green fluorescent protein (cpsfGFP). Conformational changes upon binding of 5-HT initiate a change of environment around the chromophore and lead to increased GFP fluorescence intensity. (**B**) HEK293MSR cells stably transfected with the biosensor (HEK293MSR_iSeroSnFR cells) express the 5-HT biosensor in the cytoplasm and can be transiently transfected with a monoamine transporter of choice. Transport of 5-HT into the cell via the membrane transporter and subsequent binding of 5-HT to the biosensor results in an increased GFP signal (influx mode). Adding a releasing drug to HEK293MSR_iSeroSnFR cells preloaded with 5-HT leads to 5-HT transport out of the cell (efflux) and thus a decrease in GFP signal (efflux mode). (**C**) Influx mode of the assay that can be used to determine K_m_ and V_max_. Addition of neurotransmitter (NT) to HEK293MSR_iSeroSnFR cells expressing a monoamine transporter leads to a NT concentration dependent increase in fluorescence over time. Plotting the fluorescence intensity measured after a certain incubation time as a function of the NT concentration results in an uptake curve with Michaelis–Menten kinetics. (**D**) Influx mode of the assay used to determine inhibitory potencies. Pre-incubation of HEK293MSR_iSeroSnFR cells expressing a monoamine transporter with increasing inhibitor concentrations and subsequent addition of a constant concentration of NT leads to an increased fluorescence over time negatively dependent on inhibitor concentrations. Plotting the fluorescence intensity measured after a certain incubation time as a function of the inhibitor concentration, results in a classical sigmoidal dose–response curve. (**E**) Efflux mode of the assay used to determine drug-induced neurotransmitter release. Pre-loading of HEK293MSR_iSeroSnFR cells expressing a monoamine transporter with NT in a saturating concentration and subsequent addition of increasing concentrations of a NT releasing drug leads to a drug concentration dependent decrease in fluorescence over time. Plotting the fluorescence change (corresponding to the slope of the fluorescence decrease) as a function of drug concentrations, results in an efflux curve, where the potency of the releaser can be determined.

We set out to develop a fluorescence-based assay that enables and simplifies the quantification of 5-HT transport mediated by the high-affinity, low-capacity human SERT (hSERT) as well as the low-affinity, high-capacity OCT2 without the use of radiolabeled substrates. We sought to establish an assay that would be homogenous and in real-time be able to report both influx and efflux of 5-HT by means of changes in fluorescence intensity of the genetically encoded 5-HT biosensor iSeroSnFR^30^ in a version localized to the cytoplasm. The biosensor is expected to monitor changes in cytosolic concentrations of 5-HT (Fig. 1, Fig. S2) and should therefore be able to report on both transporter-mediated influx of 5-HT applied to the outside of the cell (influx mode, Fig. 1A,B) and the subsequent transporter-mediated efflux of 5-HT loaded into the cytosol (efflux mode, Fig. 1A,B). In addition, the homogenous nature of the assay should allow repeated fluorescence measurements of the same well, negating the need for numerous parallel wells for normalization or control measurements as it is needed in conventional radiotracer assays, which are end-point assay relying on washing and termination of the transport process before quantification of radioactivity. An outline of how the 5-HT_FAsTR assay is expected to work is shown for determination of key kinetic parameters of transport (Fig. 1C), potency of transporter inhibitors (Fig. 1D) and potency and efficacy of amphetamine-like releaser compounds (Fig. 1E).

For simplicity, convenience and consistency in subsequent experiments we sought to establish a cell line stably expressing the cytoplasmic version of the genetically encoded fluorescent 5-HT biosensor iSeroSnFR developed by Unger et al.^30^. The biosensor iSeroSnFR is a hybrid of the periplasmic binding protein (PBP) OpuBC from *Thermoanaerobacter sp. X513* and a circular permutated superfolder green fluorescent protein (cpsfGFP) that increases its fluorescence intensity upon 5-HT binding to the modified PBP (Fig. 1A).

We inserted the cytoplasmic version of iSeroSnFR into the bicistronic pIRES vector containing the iSeroSnFR gene in multiple cloning site (MCS) A and a blasticidin resistance gene in MCS B, assuring that both genes are expressed from the same mRNA, ensuring stringent negative selection of cells not expressing the biosensor. We then transfected HEK293MSR cells with the pIRES_iSeroSnFR_BlasR construct and after a short selection period with blasticidin, the cells were sorted based on their GFP signal by the use of fluorescence activated cell sorting (FACS). Cells showing a GFP signal and thus robust iSeroSnFR expression were collected and cultured. HEK293MSR_iSeroSnFR cells were subsequently monitored for their biosensor expression by FACS once a week and generally showed > 90% GFP-positive cells.

While the proportion of stable biosensor cells remained very high we consistently observed that enrichment of the cell line by selecting the fraction containing high expressing cells was not long lasting (data not shown), indicating that the subpopulation with high expression levels of the 5-HT biosensor was subject to harsh negative selection via reduced growth rates and/or viability. The iSeroSnFR expression level that the stably transfected culture then stabilized at with moderate subsequent blasticidin selection resulted in a stable and still, highly fluorescent cell line that proved useful in the assay measuring 5-HT influx and efflux.

**Figure 2:**
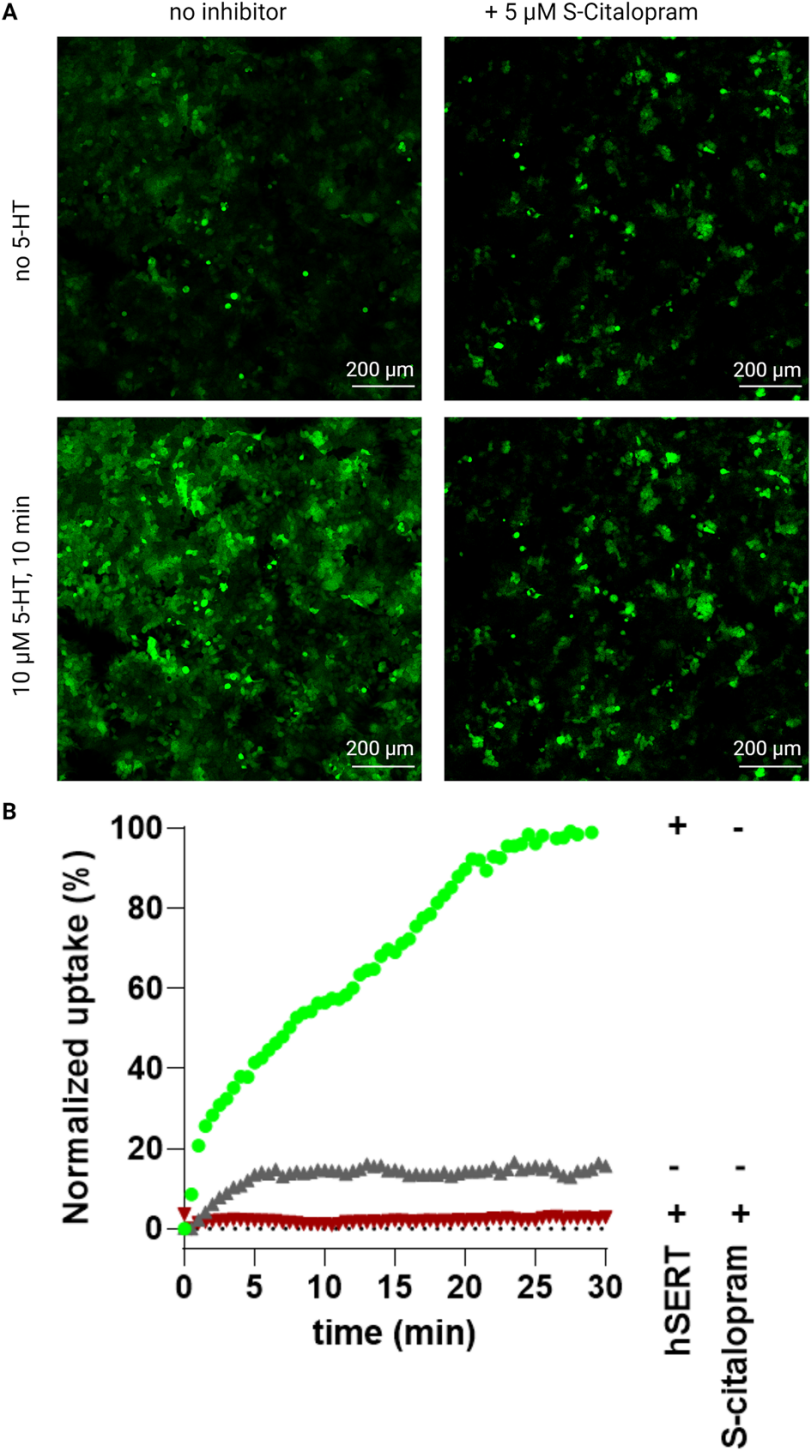
Fluorescence microscopy of HEK293MSR_iSeroSnFR cells. (**A**) Microscopy images of HEK293MSR_iSeroSnFR cells expressing hSERT before (top) and after (bottom) incubation with 10 µM 5-HT for 10 min. For control experiments, hSERT was blocked with 5 µM S-Citalopram for 15 min or HEK293MSR_iSeroSnFR cells were mock transfected. (**B**) Normalized fluorescence changes over time as a result of 5-HT uptake measured with the fluorescence microscope. Green points represents the fluorescence signal from fluorescent biosensor cells also expressing hSERT, whereas red points represent the nonspecific uptake in fluorescent biosensor cells expressing hSERT but inhibited with S-Citalopram. Grey points represent the negative control from fluorescent biosensor not expressing hSERT. Images were analysed in ImageJ by using built-in threshold algorithms on the image stacks.

In HEK293MSR_iSeroSnFR cells stably expressing the biosensor and co-expressing hSERT we observed, using confocal fluorescence microscopy, that addition of 5-HT caused an increase in fluorescence over time (Fig. 2). Control experiments with HEK293MSR_iSeroSnFR cells not expressing SERT or cells with the transporter inhibited selectively by S-Citalopram (Fig. 2) both resulted in no change of fluorescence over time and show that the observed increase in fluorescence is consistent with 5-HT uptake by SERT.

### The 5-HT_FAsTR fluorescence-based uptake assay reproducibly captures key functional aspects of serotonin transporter mediated serotonin uptake

With the stable cell line proving successful in fluorescence microscopy we rationalized that the principle could be transferred to a microplate setup for high throughput functional assays of 5-HT uptake. In this assay, uptake (influx) of 5-HT via its membrane transporter hSERT will result in increased cytoplasmic concentrations of 5-HT which in turn would yield an increased fluorescence intensity signal from the biosensor in cells expressing iSeroSnFR (Fig. 1B,C). Similar to radiotracer influx studies this increase in fluorescence signal, representing 5-HT uptake, will depend on the extracellular 5-HT concentration in a manner consistent with Michaelis–Menten kinetics for the serotonin transporter-mediated uptake process. If the fluorescence-based assay is performing as intended, it should yield kinetic parameters similar to that obtained in radiotracer uptake assays.

**Figure 3:**
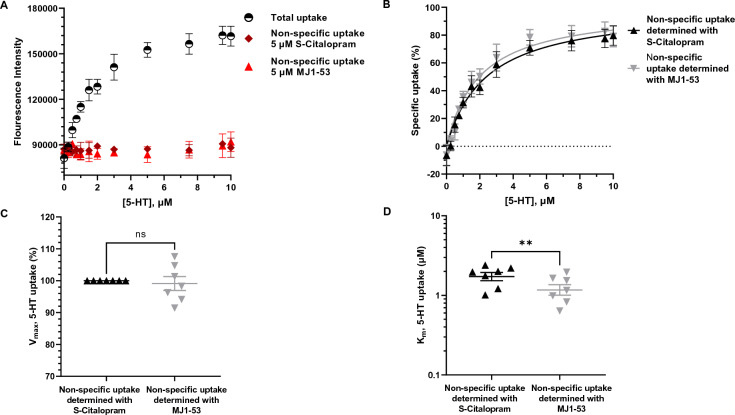
5-HT_FAsTR reliably determines transport kinetic parameters in a high throughput 96-well microplate setup. (**A**) Graph showing fluorescence intensity in response to increasing concentrations of unlabeled 5-HT added to HEK293MSR_iSeroSnFR cells in a representative experiment. Shown is the total uptake of 5-HT (black, half open circles) and the non-specific uptake of 5-HT when blocking hSERT with the SSRI S-Citalopram (dark red diamonds) and when blocking hSERT with the membrane-impermeant imipramine analogue MJ1-53 (light red triangles). Shown are mean ± SD of four technical replicates for the total uptake and technical duplicates for the non-specific uptake. Each technical replicate constitutes the mean of four measurements conducted at four different positions in the same microplate well after 10 min incubation with 5-HT. (**B**) Representative graph showing the specific uptake of 5-HT via hSERT normalized to the maximal uptake calculated from A). Each point show the specific uptake calculated by subtracting either nonspecific uptake using S-citalopram (black traingles) or MJ1-53 (grey inverted triangles) from total uptake values. Shown are mean ± SD of four replicates and a Michaelis–Menten fit with robust regression. (**C**) Comparison of V_max_ values determined by a Michaelis–Menten fit shows no difference between the inhibitor used to determine non-specific uptake. Shown are mean ± SEM from seven independent experiments. D) MJ1-53 appears to yield slightly lower KM values than S-Citalopram when used as a hSERT inhibitor to determine non-specific uptake. Shown is a comparison of mean K_M_ values ± SEM from seven independent experiments determined by a Michaelis–Menten fit using either S-Citalopram or MJ1-53 to determine non-specific uptake. Statistical analysis of the difference is performed with a paired t-test (**P = 0.0032).

**Figure 4:**
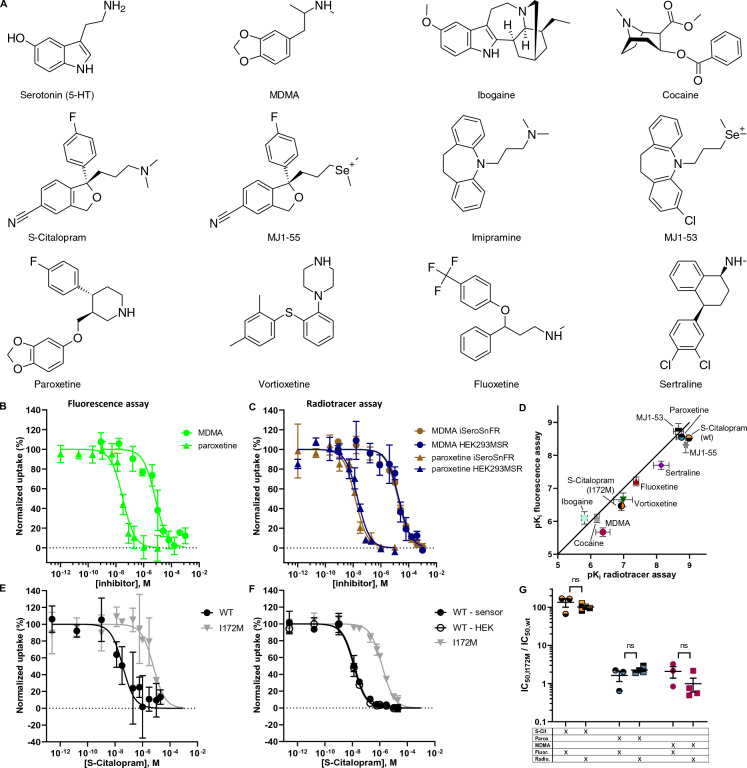
The performance of 5-HT_FAsTR in uptake inhibition assays is comparable to radiotracer uptake inhibition assays. (**A**) Chemical structures of hSERT substrates and inhibitors. (**B**) and (**C**) Representative graphs showing normalized 5-HT uptake in response to increasing MDMA (circle) and paroxetine (triangle) concentrations in (**B**) the fluorescence assay and (**C**) the radiotracer assay. Shown is the normalized uptake of 5-HT in HEK293MSR_iSeroSnFR cells stably expressing the biosensor (green or brown) and HEK293MSR cells not expressing the biosensor (blue). Shown are mean ± SD of duplicates for MDMA and four replicates for paroxetine and a sigmoidal dose–response fit using non-linear regression. (**D**) Comparison of pK_i_ values of 10 hSERT inhibitors determined with the fluorescence assay versus pK_i_ values determined with the radiotracer assay. Each data point represents the mean ± SD of three to five individual IC_50_ experiments. (**E**) and (**F**) Representative graph of normalized 5-HT uptake in response to increasing S-Citalopram concentrations in (**E**) the fluorescence assay and (**F**) the radiotracer assay. Shown is the normalized uptake of 5-HT in HEK293MSR_iSeroSnFR cells expressing either hSERT wild type (wt) (black circles) or the hSERT mutant I172M (grey triangles). Shown are mean ± SD of at least duplicates. (**G**) IC_50_ ratios for hSERT wt and the transporter variant I172M in response to S-Citalopram, paroxetine and MDMA were found to be identical when IC_50_ values were obtained in both the fluorescence and the radiotracer assay. Shown are mean ± SEM, N = 3, unpaired t-test.

Indeed, in stably expressing HEK293MSR_iSeroSnFR cells transiently transfected with hSERT we observed specific serotonin transporter mediated 5-HT uptake as evidenced by fluorescence intensity that saturated with increasing extracellular 5-HT concentrations consistent with Michaelis–Menten kinetics (Fig. 3B). The uptake remained linear for 20 min (Fig. 2B and Fig. S1) and for the subsequent assays 10 min incubation was used to assay 5-HT uptake, similar to previous optimizations of radiotracer assays in the Sinning lab. Control experiments using the specific, high-affinity inhibitors of the serotonin transporter, S-Citalopram and MJ1-53 (a membrane-impermeant imipramine analogue), showed that blocking the serotonin transporter abolishes 5-HT uptake as measured by fluorescence intensity (Fig. 3A). Fluorescence levels at V_max_-conditions for the serotonin transporter consistently yielded a doubling of the initial basal fluorescence and a mean apparent affinity (K_m_ ± SEM) of 1.79 ± 0.19 µM (N = 7) when the selective serotonin reuptake inhibitor (SSRI) S-Citalopram was used to determine non-specific uptake (Fig. 3C,D). A mean apparent affinity (K_m_ ± SEM) of 1.25 ± 0.48 µM (N = 7) was obtained when MJ1-53 was used to determine non-specific uptake (Fig. 3C,D). The obtained K_m_ values are comparable to earlier radiotracer experiments in our laboratory and the literature^31,32^, however K_m_ values were found to be slightly different and statistically significantly higher (*P* = 0.0394) when S-Citalopram was used to determine the non-specific uptake compared to MJ1-53. We observed that high concentrations of S-Citalopram and other drugs are causing an increase in iSeroSnFR signal independent of 5-HT. We hypothesized that at high inhibitor concentrations diffusion of the drug through the cell membrane may lead to a subsequent change of pH in the cytosol that may change iSeroSnFR fluorescence intensity independent of 5-HT. When expressing iSeroSnFR on the membrane we observed that iSeroSnFR is indeed pH dependent as many GFP-species are and that fluorescence increases with increasing pH (data not shown). This is consistent with the hypothesis that diffusion of the weak base S-Citalopram into the cytoplasm increases cytoplasmic pH and as a result iSeroSnFR fluorescence, which may lead to subtle overestimation of non-specific 5-HT uptake. Since the imipramine analogue MJ1-53 is permanently positively charged (Fig. 4A), it cannot cross the cell membrane while still maintaining high potency for inhibition of SERT and thus provides more reliable kinetic parameters when high drug concentrations are necessary.

Collectively these data demonstrate that the fluorescence-based assay enables us to reproducibly determine the relative maximal uptake capacity (V_max_) of hSERT as well as 5-HTs apparent affinity (K_m_) for hSERT and yields kinetic parameters similar to that obtained in radiotracer uptake assays.

### The 5-HT_FAsTR fluorescence-based uptake assay is useful in high-throughput pharmacological studies of serotonin transporter inhibitors

Having shown that the fluorescence-based assay enables us to study 5-HT uptake, we next sought to explore if the same setup is suitable for a high throughput drug screen by reproducing reliable inhibitory potencies (pK_i_ values) of known hSERT inhibitors. We rationalized that concentration dependent inhibition of 5-HT uptake via its membrane transporter hSERT will result in a concentration dependent decrease of cytoplasmic 5-HT concentrations which in turn would result in a decrease of fluorescence signal from the biosensor in cells expressing iSeroSnFR (Fig. 1B,D). Similar to radiotracer influx studies, a decrease in fluorescence signal, representing the inhibition of 5-HT uptake, will depend on the extracellular inhibitor concentration and yield a sigmoidal dose–response curve representing the inhibition of serotonin transporter-mediated uptake (Fig. 1D). If the fluorescence-based assay is performing as intended, it should yield inhibitory potencies similar to those obtained in radiotracer assays.

We tested known hSERT inhibitors (structures are shown in Fig. 4A) in the fluorescence-based assay and indeed observed a decrease in the fluorescence signal in response to increasing inhibitor concentrations, reflecting the inhibition of serotonin transporter mediated 5-HT uptake (Fig. 4B–D). When optimizing the fluorescence-based assay, we found a 5-HT concentration of 10 µM to be optimal for studying uptake inhibition. To be able to compare the obtained IC_50_ values to inhibitory potencies determined with radiotracer uptake assays in literature, we converted these to pK_i_ values using the Cheng-Prusoff equation^33^. As mentioned above, we observed a 5-HT independent increase in iSeroSnFR signal when incubating HEK293MSR_iSeroSnFR cells with high concentrations of drugs that are able to cross the cell membrane and thus might cause a subsequent change of pH in the cytosol. Therefore, we decided to use the membrane impermeant, permanent positively charged MJ1-53 (20 µM) (for structure see Fig. 4A) to determine the bottom plateau of the inhibition curve (non-specific uptake) where high concentrations of membrane permeant drugs might interfere with the biosensor. In theory, binding of 5-HT to the intracellular biosensor could act as a sink for the translocated 5-HT and affect the transport process. However, Fig. 4C shows, that pK_i_ values obtained in the radiotracer flux assay are virtually identical for HEK293MSR_iSeroSnFR cells and HEK293MSR cells not expressing the biosensor, proving that the biosensor itself does not influence the uptake process but as desired it passively monitors the cytoplasmic levels of 5-HT. Next, we determined IC_50_ values for a range of known hSERT inhibitors in the conventional radiotracer assay and the new fluorescence-based label-free assay in parallel and obtained comparable pK_i_ values for all tested inhibitors (Fig. 4D). Collectively we observe very high correlation between the fluorescence-based assay and the radiotracer assay. Our data demonstrate that the developed fluorescence assay allows us to determine inhibitory potencies of hSERT inhibitors without the specialized laboratory facilities and equipment associated with radioisotope protocols.

After establishing how the 5-HT_FAsTR fluorescence-based assay can be used to determine inhibitory potencies of drugs targeting hSERT, we sought to test if the assay would be useful for studying the impact of mutations or genetic polymorphisms on i.e. inhibitory potencies. We chose the hSERT_I172M mutation in the transmembrane domain 3 because this mutation would pressure-test how well 5-HT_FAsTR handles both large and small shifts in inhibitory potencies. Henry et al. showed that this mutation of hSERT selectively impacts the binding of the SSRI S-Citalopram tremendously, while the potency of the SSRI paroxetine for this mutant remains almost unchanged^34^. It is still unclear from biochemically validated models and subsequent structures why I172M so acutely distinguishes between S-citalopram and paroxetine because for both compounds the sidechain of I172 is located in the crevice between the two aromatic systems in a similar fashion^35–37^, but this effect has been replicated in multiple laboratories. Indeed, we consistently obtain similar fold changes in both the newly developed 5-HT_FAsTR fluorescence-based assay and the conventional radiotracer assay. As expected, we observed a marked loss of inhibitory potency for the SSRI S-Citalopram in the I172M mutant, while the mutation I172M did not show any change in the potencies of the SSRI paroxetine and the stimulant MDMA (Fig. 4E–G). While Henry et al. observed a 752-fold decrease in the potency of S-Citalopram towards hSERT I172M compared to the wild type (wt), we only observed a 135-fold decrease in the fluorescence assay and a 102-fold decrease in the radiotracer assay (Fig. 4G), which is also consistent with Rannverson et al.^38^. The minor fold difference between the 5-HT_FAsTR and radiotracer assay is not statistically significant. For paroxetine we observed a 1.6-fold and 2.6-fold decrease in potency in the fluorescence and the radiotracer assay, respectively, which is not statistically significant and also corresponds well to the decrease in potency identified by Henry et al. (2.1-fold)^34^ and Davis et al. (2.3-fold)^36^. Thus, 5-HT_FAsTR allows us to detect a wide spectrum of changes in the molecular pharmacology of antidepressants in hSERT mutations.

### The 5-HT_FAsTR fluorescence-based uptake assay is useful for efflux studies and pharmacological characterization of amphetamine-like releaser-type compounds

**Figure 5:**
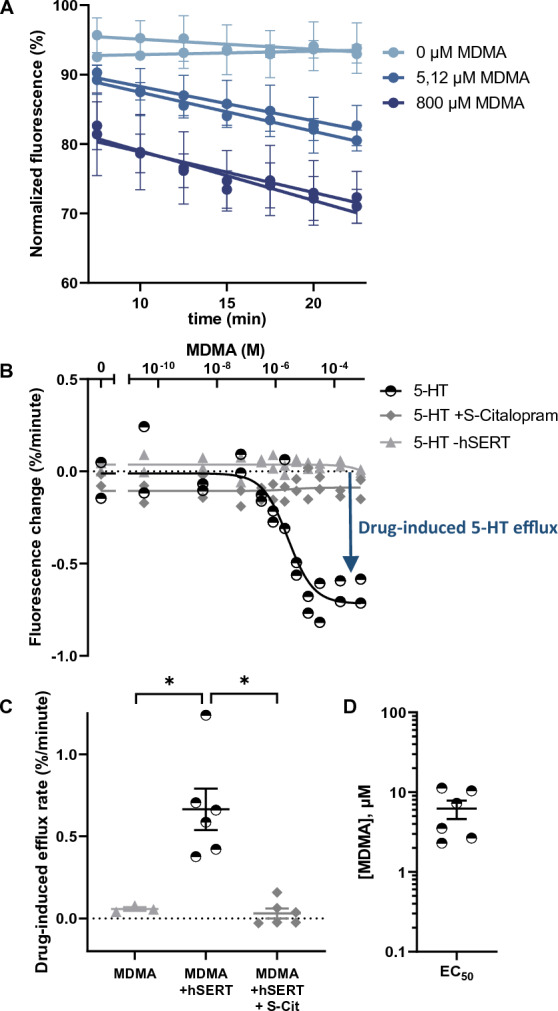
5-HT_FAsTR fluorescence-based efflux assay to characterize ecstasy (MDMA) action on the serotonin transporter. (**A**) Representative graph showing the change in normalized fluorescence in response to three different MDMA concentrations over time. MDMA was added at time point 0 and a time window showing efflux in the linear range was chosen for further analysis. The fluorescence intensity measured in response to MDMA was normalized to the mean fluorescent intensity measured 2.5 min, 5 min and 7.5 min before the addition of MDMA. The response to MDMA was measured in duplicates at four different positions in each well. Shown are mean ± SD of four measurements per well for both duplicates and a simple linear regression performed with GraphPad Prism. (**B**) Representative dose–response curve for efflux induced by MDMA. The slope of the linear decrease in fluorescence in response to MDMA, calculated with the simple linear regression shown in (**A**), was plotted as a function of increasing MDMA concentrations added to the HEK293MSR_iSeroSnFR cells. A non-linear regression fit to a sigmoidal dose–response curve was used to calculate EC_50_, the top and the bottom plateau. Shown is the efflux rate in response to MDMA for cells transfected with hSERT and preloaded with 5-HT (black, half open circles), transfected with hSERT, preloaded with 5-HT and incubated with the hSERT inhibitor S-Citalopram (dark grey squares) or transfected with an empty vector and preloaded with 5-HT (light grey triangles). (**C**) Maximum drug-induced efflux rate of six individual experiments, calculated by subtracting the top plateau from the bottom plateau of the dose–response curve in B). Shown are mean ± SEM, N = 6, *P = 0.037 for MDMA vs. MDMA + hSERT and *P = 0.017 for MDMA + hSERT vs. MDMA + hSERT + S-Cit, one-way Anova with multiple comparisons (Tukey test). (**D**) EC_50_ values for efflux induced by MDMA from six individual experiments calculated with a non-linear fit as shown in figure B. Shown are mean ± SEM.

Some amphetamine-like drugs act by inhibiting monoamine transporters like hSERT, however most amphetamine analogs exert their effect by initiating the release of neurotransmitters via their membrane transporters, most likely via an exchange mechanism. We examined whether the new 5-HT_FAsTR fluorescence-based assay could be used as an approach to detect transporter-mediated 5-HT release. We pre-loaded HEK293MSR_iSeroSnFR cells with unlabeled 5-HT and rationalized that drug-induced efflux of 5-HT via its membrane transporter hSERT will result in a releaser concentration dependent decrease of cytoplasmic 5-HT concentrations which in turn would result in a decrease of fluorescence signal from the biosensor (Fig. 1B,E). Indeed, MDMA elicited a dose-dependent decrease in intracellular 5-HT levels, measured as a decrease in GFP-fluorescence, in hSERT-transfected HEK293MSR_iSeroSnFR cells (Fig. 5A). The relative release rate or efflux rate is defined by the decrease rate in fluorescence. Plotting the slope of the observed fluorescence decrease as a function of the MDMA concentration (Fig. 5B) resulted in a maximum fluorescence change ± SEM of 0.66 ± 0.13%/minute (N = 6). Importantly, when hSERT was either not present or was blocked by the SSRI, S-Citalopram, we detected no fluorescence change (fluorescence change ± SEM of 0.06 ± 0.01%/minute (N = 3) and 0.03 ± 0.03%/minute (N = 6), respectively) (Fig. 5C).

This suggests that the observed release of 5-HT caused by MDMA is at least 11-fold higher than background levels from passive diffusion and that this MDMA-induce release occurs via reverse transport (efflux) by hSERT, consistent with the literature describing MDMA as an efflux inducing drug for SERT^39^. Our measured relative release rate is lower than that described using radiolabeled 5-HT in a superfusion setup^40^, which we ascribe to differences in cell type and the fact that the superfusion study is performed after a wash-out period with resulting lower intracellular 5-HT concentrations and thus a higher proportion of the remaining neurotransmitter release carried by SERT, whereas we sample release rates at a time point where the intracellular concentrations of 5-HT are higher and the limited turn-over rate of SERT becomes more pronounced relative to passive diffusion. Dose–response curves obtained from six individual experiments provided a releasing potency (EC_50_ ± SEM) for MDMA of 6.22 ± 1.62 µM (Fig. 5D), comparable to the literature^40^ and suggests that the fluorescence assay in efflux mode is capable of not only discerning releasers from inhibitors but also capable of measuring potencies for releasers, e.g. of the amphetamine class. Collectively these data demonstrate that the 5-HT_FAsTR fluorescence-based efflux assay detects changes of 5-HT concentrations inside the cell with a high temporal resolution and is able to quantify transporter mediated 5-HT release. The fluorescence-based assay is thus able to determine both uptake of 5-HT via hSERT, inhibitory potencies of hSERT inhibitors and the releasing effect of efflux inducing drugs and hereby shows potential to be applied as a screening tool to determine the potency of drugs with the ability to also distinguish inhibitors from releasers.

### The 5-HT_FAsTR fluorescence-based uptake assay can be applied to study serotonin transport via low-affinity, high-capacity transporters of the Organic Cation Transporter family

**Figure 6:**
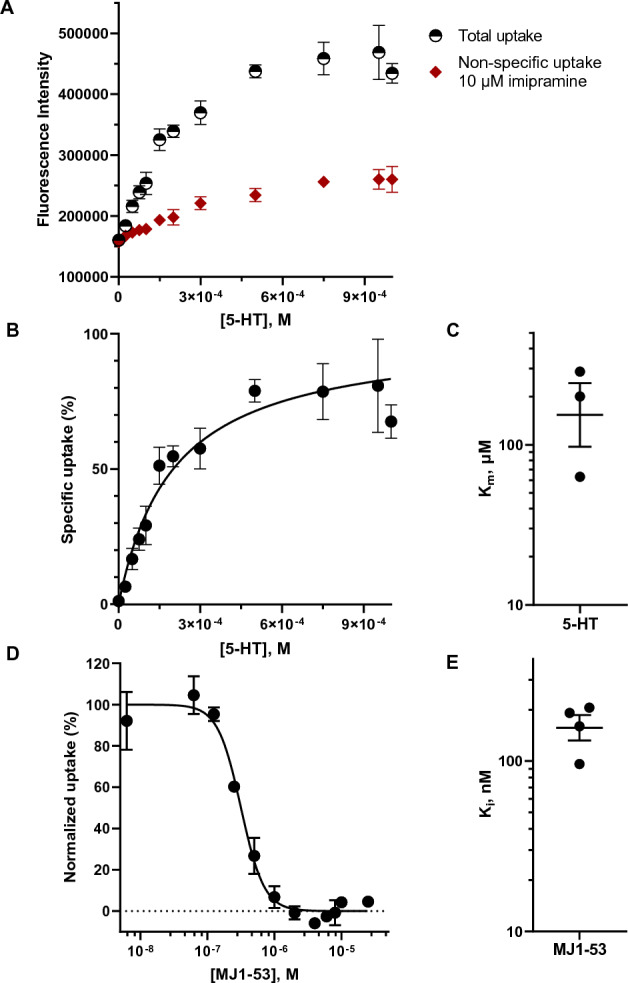
5-HT_FAsTR can be used to quantify uptake and pharmacology of a high-capacity, low-affinity organic cation transporter. (**A**) Representative graph showing fluorescence intensity in response to increasing concentrations of unlabeled 5-HT added to HEK293MSR_iSeroSnFR cells transfected with hOCT2. Shown is the total uptake of 5-HT (black, half open circles) and the non-specific uptake of 5-HT when blocking hOCT2 with the TCA imipramine (dark red squares) as mean ± SD of four replicates. Each replicate constitutes the mean of nine measurements conducted at different positions in the same well. (**B**) Representative graph showing the specific and saturable uptake of 5-HT via hOCT2 normalized to the maximal uptake. Shown are mean ± SD of four replicates and a Michaelis–Menten fit using non-linear regression. (**C**) K_m_ values determined for three independent experiments by a Michaelis–Menten fit with robust regression, shown are mean ± SEM. (**D**) Representative graph showing normalized 5-HT uptake via hOCT2 in response to increasing concentrations of the imipramine analogue MJ1-53. Shown are mean ± SD of duplicates and a non-linear fit ([inhibitor] vs response variable slope – (four parameters). (**E**) K_i_ values of four independent experiments, shown are mean ± SEM.

5-HT is not only transported across cell membranes by the high-affinity, low-capacity monoamine transporter hSERT but also by the promiscuous low-affinity, high-capacity organic cation transporters (OCTs) and plasma membrane monoamine transporter (PMAT). As mentioned above, OCTs are targets for amphetamine-based designer drugs, but OCTs are furthermore known to play an important role in therapeutic drug import and export. Understanding how therapeutics are accumulated or ejected from cells via OCTs thus potentially sheds new light on inter-patient differences in response to medicine. We rationalized, that the 5-HT_FAsTR fluorescence-based uptake assay can be used to monitor the activity of any transporter that is able to facilitate 5-HT transport via the cell membrane. If this is the case, the uptake (influx) of 5-HT via the human organic cation transporter 2 (hOCT2) will result in increased cytoplasmic concentrations of 5-HT and yield an increased fluorescence signal from the biosensor similar to hSERT. Indeed, we observed a concentration dependent increase of specific 5-HT uptake (Fig. 6A,B) and obtain a mean apparent substrate affinity (K_m_ ± SEM) of 183.6 ± 65.18 µM (N = 3) (Fig. 6C) when the TCA imipramine was used to determine non-specific uptake, comparable to values in the literature^41,42^. We furthermore rationalized, that a decrease in fluorescence signal, representing the inhibition of 5-HT uptake via hOCT2, will depend on the extracellular inhibitor concentration and yield a sigmoidal dose–response curve representing the inhibition of hOCTs-mediated uptake. As expected, we observed that inhibition of hOCT2 with the imipramine analogue MJ1-53 resulted in an inhibitor-dependent decrease in fluorescence and yielded a pK_i_ ± SEM of 6.8 ± 0.07 (N = 4), corresponding to a K_i_ of 163.6 nM, in the fluorescence-based assay (Fig. 6D,E).

Collectively, these data show, that the 5-HT_FAsTR fluorescence-based assay is not only useful for studying uptake, uptake inhibition and efflux via the high-affinity, low-capacity membrane transporter hSERT, but can also be applied to a low-affinity, high-capacity serotonin transporter like hOCT2. We conclude that the versatility of 5-HT_FAsTR allows us to functionally and pharmacologically characterize different serotonin transporters, and mutants thereof, with very different properties.

## Discussion

For decades, the study of serotonin transporters primarily relied on radioisotope-based methods with all the practical complexities that it entails. Here we developed and validated a versatile and cost-effective tool that in most laboratories with standard cell culture capabilities and a fluorescence plate reader can be used both for high-throughput screening purposes and for detailed functional characterization of various serotonin transporters and mutants thereof. The method allowed us to both characterize antidepressants acting as inhibitors of monoamine transporters and to distinguish between the inhibitory and the releasing effect of the amphetamine-like drug MDMA (“ecstasy”) on the serotonin transporter. The stable, intracellular expression of the biosensor enables the direct detection of 5-HT transported into the cytosol and constitutes a major advantage of 5-HT_FAsTR over other methods that utilize GPCR-based biosensors that by design can only measure extracellular neurotransmitter. Another advantage of the fluorescence assay is that opposed to radiotracer studies, which are end point assays requiring termination, washing, cell lysis and scintillation cocktails, 5-HT_FAsTR can repeatedly measure changes in intracellular 5-HT concentrations essentially in real-time without termination of the assay. Aside from decreasing the work load associated with performing the assay and decreasing the consumption of materials, it also has the advantage of not requiring tedious optimization of time courses etc. because continuous quantification of the changes in intracellular 5-HT over time allows the experimenter to choose the appropriate incubation time after the experiment has been performed. In many settings the continuous measurement of the same well in the fluorescence-based assay can be used for normalization of a response after an intervention in that well, e.g. efflux before and after MDMA, which in the radiotracer assay would require multiple parallel wells for normalization with the inevitable well-to-well variation that inflicts on the radiotracer data.

We show that the 5-HT_FAsTR live cell assay can be applied to study the effect of antidepressants on the hSERT wt as well as transporter variants and that the assay furthermore is applicable to study uptake and uptake inhibition of 5-HT via the low-affinity, high-capacity organic cation transporter hOCT2. Study of OCTs with isotopes can be problematic and costly because they require high 5-HT concentrations with substantial consumption of isotope as well as issues with high non-specific binding of 5-HT. 5-HT_FAsTR only measures intracellular 5-HT and is radiotracer-free, thereby negating the practical problems associated with studying 5-HT uptake via low-affinity transporters where a high 5-HT concentration is needed. It is also noteworthy how well the fluorescence-based assay straddles both extremes of serotonin transporters much more effortlessly than the radiotracer uptake assay; both the high-affinity, low-capacity SERT and the low-affinity, high-capacity OCT2 are robustly assayed.

A limitation of the method arises from the pH sensitivity of the 5-HT biosensor iSeroSnFR, which may be an issue when combined with high concentrations of the partially membrane permeable drugs and medications targeting the serotonin transporter. Because virtually all serotonin transporter inhibitors are amines, they can diffuse across the membrane in the unprotonated form and act as weak bases in the cytoplasm. As observed in both the 5-HT_FAsTR uptake and uptake inhibition assay, high concentrations of the tested drugs caused an artificial increase in the fluorescence signal. Further investigations of the effect of high drug concentrations on intracellular pH would shed light on the observed phenomenon and the development of a pH insensitive biosensor could solve the observed limitation. However, we also find that permanently positively charged antidepressant analogs can be used to determine non-specific transport rates at full inhibition levels, which allows us to use drug concentrations of the inhibitors that are sufficient to determine the IC_50_ reliably even for drugs with only moderate potency but not be forced to use excessive amounts that may affect the intracellular pH and resultingly the serotonin biosensor. The intracellular localization of the biosensor and thus its read-out being a negative change in fluorescence signal, constitutes a direct measure of cytoplasmic 5-HT levels, albeit an indirect detection of 5-HT release via the serotonin transporter. However, we show that the same experimental set up used to study uptake and uptake inhibition can be applied to study efflux of 5-HT induced by releasing agents. In our hands the 5-HT_FAsTR efflux assay is able to quantify drug-induced efflux and provides a reliable potency of the amphetamine-like drug, MDMA, for inducing efflux.

To the best of our knowledge, the 5-HT_FAsTR assay is the first of its kind differentiating 5-HT releasers from SERT inhibitors that is both radiotracer-free, has a high temporal resolution, detects changes of 5-HT concentrations inside the cell and has high throughput useful for drug screening campaigns. The assay shows potential to facilitate and accelerate the classification of new 5-HT releasers based on direct measurement of their pharmacological action in a high-throughput format and hereby constitutes a promising tool to guide regulatory agencies in assessing how new designer drugs should be classified and controlled. 5-HT_FAsTR is also able to take the place of radiotracer assays in most, if not all, functional studies of serotonin transporters and mutants thereof. 5-HT_FAsTR democratizes the study of serotonin transporters as a general tool accessible to most laboratories for studying disease mutations, for example mutations of monoamine transporters linked to psychiatric disorders, and their influence on transporter function. Additionally, the assay has great potential for becoming a general tool to screen new pharmaceuticals for treatment against psychiatric disorders like ADHD and depression, where 5-HT releasing agents and serotonin transporter inhibitors are applied as treatment, respectively. The plasmid (Addgene) and cell line (contact the corresponding author) used for 5-HT_FAsTR is available to interested researchers.

### Material and data availability

The plasmid for stable transfection with cytoplasmic iSeroSnFR in pIRES vector is available from Addgene (Plasmid #214132). Stable HEK293MSR cells expressing cytoplasmic iSeroSnFR from the pIRES vector and data is available from the corresponding author on request.

## Materials and methods

### Cell culture

*HEK293MSR-iSeroSnFR cells* Human Embryonic Kidney 293 cells expressing the macrophage scavenger receptor (HEK293MSR, Invitrogen) for better adherence to plastic surfaces and the biosensor iSeroSnFR^30^ were cultured in DMEM high glucose (Gibco or Biowest) containing 10% FBS (Sigma), 100 units/ml penicillin and 0.1 mg/ml streptomycin (Gibco or Sigma-Aldrich), 0.1 mM MEM Non-Essential Amino Acids (MEM NEAA; Gibco or Biowest), 0.6 mg/ml Geniticin (ChemCruz) and 6 µg/ml Blasticidin (Fisher Scientific) at 37 °C and 5% CO_2_. Cells were passaged every 2–3 days by removing the growth medium, washing them in PBS (Ca/Mg^2+^ free) and 0.48 mM versene and detaching them using 1× trypsin–EDTA (Sigma).

*Stable cell line generation* Polyclonal cell line. HEK293MSR cells were transfected with linearized plasmid DNA of an pIRES vector containing the gene encoding iSeroSnFR^30^ in multiple cloning site (MCS) A and the gene encoding blasticidin resistance in MCS B. pIRES_iSeroSnFR_Blas was digested with Bgl II (Thermo Fisher Scientific) and transfection was performed as described below using EcoTransfect (OZ Biosciences). After selection with 8 µg/ml blasticidin for 1 week, the cells were sorted based on their expression levels of the green fluorescent biosensor iSeroSnFR via fluorescent activated cell sorting (FACS) in the FACS Core Facility, Aarhus University, Denmark. Sorting was repeated three times with an interval of 25 and 10 days respectively. Monoclonal cell line. Single cell sorting of HEK293MSR_iSeroSnFR cells with high sensor expression was performed to obtain monoclonal cell lines with either low, medium or high sensor expression. Cells were collected in a flat bottom clear, black polystyrene 96 well plate with a tissue culture-treated surface (Corning) containing DMEM high glucose (Gibco or Biowest) containing 10% FBS (Sigma), 100 units/ml penicillin and 0.1 mg/ml streptomycin (Gibco or Sigma-Aldrich), 0.1 mM MEM Non-Essential Amino Acids (MEM NEAA; Gibco or Biowest), 0.6 mg/ml Geniticin (ChemCruz) and 0.6 mg/ml Blasticidin (Fisher Scientific) and cultured at 37°C and 5% CO_2_.

*Flow cytometry and fluorescent activated cell sorting (FACS)* Flow Cytometry and FACS was performed in the FACS Core Facility, Aarhus University, Denmark. HEK293MSR_iSeroSnFR cells were detached from the culture flask as described above. For flow cytometry analysis, 2.5 × 10^6^ cells were centrifuged at 100 g for 10 min and re-suspended in 250 µl PBS (Ca^2+^/Mg^2+^ free) supplemented with 0.5% FBS (Sigma). For sorting, 10 × 10^6^ cells were centrifuged at 100 g for 10 min and re-suspended in 1 ml PBS (Ca/Mg^2+^ free) supplemented with 2% FBS (Sigma), 2.5 mM EDTA and 25 mM HEPES buffer pH 7. The cells were kept on ice until analysis or sorting in the FACS Core Facility, Aarhus University, Denmark. Before analysis and sorting, the cells were filtered through a sterile, syringe-type 50 µm filter (Filcon). Analysis was performed with a NovoCyte 3000 flow cytometer (Agilent, Santa Clara, CA) and sorting was performed with a FACSAria III high speed cell sorter (BD Biosciences, San Jose, CA). The NovoCyte 3000 flow cytometer was equipped with three lasers (405 nm, 488 nm and 640 nm), 13 fluorescence detectors and the NovoExpress Software version 1.5.6 (Agilent, Santa Clara, CA). The FACSAria III was equipped with 4 lasers (405 nm, 488 nm, 561 nm and 633 nm) and 12 fluorescence detectors and the BD FACSDiva Software version 8.0.2 (BD Biosciences, San Jose, CA). A 488 nm, 20 mW laser and a 488/10 filter were used to detect forward and side scatter (FWS and SSC). A 488 nm, 20 mW laser and a 530/30 filter were used to detect GFP and a 561 nm, 50 mW laser and a 610/20 filter were used to detect propidium iodide (PI) for both instruments and mock transfected cells were used as control. The FACSAria III performance was checked on a daily basis by CS&T research beads (BD Biosciences) and Accudrop Beads (BD Biosciences). Polyclonal cell line. The first FACS was an aseptic, 2-way sort with a 100 µm nozzle, a ND filter of 1.5, a purity sort mask and was performed at 4°C sample and collection temperature by the FACS core staff. Two cell populations were collected – one population with low sensor expression (1 × 10^6^ cells) and one with high sensor expression (9000 cells). Cells were collected in collection media (Ca/Mg^2+^ free PBS) supplemented with 20% FBS (Sigma), 2.5 mM EDTA, 200 units/ml penicillin, 0.2 mg/ml streptomycin and 25 mM HEPES buffer pH 7) in 5 ml tubes. Freshly sorted cells were centrifuged (100 g for 10 min), re-suspended and cultured in DMEM high glucose (Gibco or Biowest) containing 10% FBS (Sigma), 100 units/ml penicillin and 0.1 mg/ml streptomycin (Gibco or Sigma-Aldrich), 0.1 mM MEM Non-Essential Amino Acids (MEM NEAA; Gibco or Biowest), 0.6 mg/ml Geniticin (ChemCruz) and 0.6 mg/ml Blasticidin (Fisher Scientific) at 37°C and 5% CO_2_. HEK293MSR_iSeroSnFR cells were passaged every 2–3 days as described above. Both cell populations underwent a second sort 25 days later, where the 50% highest sensor expressing cells from the high sensor expressing population (1 × 10^6^ cells) and the 15% highest sensor expressing cells from the low sensor expressing population (800.000 cells) were collected and cultured as described above. Ten days later a third sort was performed on the high sensor expressing population and the 30% highest sensor expressing cells were collected and cultured as described above.

*Transfection for fluorescence and radiotracer assay* HEK293MSR cells and HEK293MSR cells expressing iSeroSnFR^30^ were transfected with the human serotonin transporter (hSERT), the human organic cation transporter 2 (hOCT2/SLC22A2, GenScript) or an empty pcDNA3.1 + vector using EcoTransfect (OZ Biosciences). Cells were transfected in flat bottom clear, black polystyrene plates with a tissue culture-treated surface (Corning). Briefly, per well 0.0625 or 0.15 µg DNA per well (hSERT and hOCT2 respectively), 16 µl FluoroBrite DMEM Media (Gibco) without serum and 2.5 µl EcoTransfect (OZ Biosciences)/µg DNA were incubated for 20 min at RT. 50 µl/well FluoroBrite DMEM (Gibco) supplemented with 10% FBS (Sigma), 100 units/ml penicillin and 0.1 mg/ml streptomycin (Gibco or Sigma-Aldrich), 0.1 mM MEM Non-Essential Amino Acids (MEM NEAA; Gibco or Biowest) and 0.6 mg/ml Geniticin (ChemCruz) was added. The cells were reverse transfected by adding the transfection mix described above and 35,000 cells in 50 µl FluoroBrite DMEM (Gibco) supplemented with 10% FBS (Sigma), 100 units/ml penicillin and 0.1 mg/ml streptomycin (Gibco or Sigma-Aldrich), 0.1 mM MEM Non-Essential Amino Acids (MEM NEAA; Gibco or Biowest) and 0.6 mg/ml Geniticin (ChemCruz) to each well. The cells were incubated for 48 h at 37 °C and 5% CO_2_ and used for the fluorescence and/or isotope assay two days post-transfection.

*Transfection for 5-HT uptake microscopy with stable cells* HEK293MSR-iSeroSnFR cells were transfected with pcDNA3-hSERT WT by following the Lipofectmaine2000 transfection reagent protocol (Invitrogen). Cells were transfected in a 35 mm glass bottom dish (#1.5, Cellvis) coated with 0.1 mg/ml poly-D-lysine (Sigma-Aldrich) dissolved in 50 mM borate buffer, pH 8.5 (Thermo Scientific). 0.6 µg DNA, 50 µl optiMEM (Gibco) and 0.9 µl Lipofectamine2000 per dish were used according to the manufacturer’s protocol. The cells were reverse transfected by adding the transfection mix described above and 600.00 cells in 2000 µl DMEM (Gibco) containing 10% FBS (Hyclone), 100 units/ml penicillin and 0.1 mg/ml streptomycin (Gibco), 1% NEAA (Gibco), 0.6 mg/ml Geniticin (Gibco) and 6 µg/ml Blasticidin (Gibco) to the dishes and incubated for 48 h at 37 °C and 5% CO_2_. Cells were imaged two days post-transfection.

### Assays

*Fluorescence plate reader assay (5-HT_FAsTR)* The fluorescence plate reader assay was conducted two days post-transfection. Briefly, cell culture media (FluoroBrite, Gibco), was removed from the wells, PBSCM (PBS supplemented with 1 mM MgCl_2_ and 0.1 mM CaCl_2_, pH 7.4) containing the substance of interest was added and the change in fluorescence was measured with either an EnSight (Perkin Elmer) or a Sense (Hidex) plate reader. The EnSight plate reader was equipped with the Kaleido software version 1.2 and a fluorescence well scan was performed, measuring fluorescence intensity at 2 × 2 positions with a distance of 1 mm in each well and at a height of 6.93 mm. The plate was excited from the bottom with a wavelength of 488 nm and emission was measured from the bottom at a wavelength of 509 nm. The number of flashes used was 100 and the whole plate was measured every 150 s. The Hidex Sense plate reader was equipped with the Hidex Sense Plate Reader Software version 1.3.0 and a fluorescence well scan was performed, measuring fluorescence intensity at 3 × 3 positions at default settings for both focus and PMT voltage. The plate was excited from the bottom with a wavelength of 485/10 nm and emission was measured from the bottom at a wavelength of 535/20 at low lamp power. The number of flashes used was 10 and the whole plate was measured every 136 s.

For the **uptake assay** determining K_m_ and V_max_ the cell culture media was removed, the cells were incubated in 40 µl pre-incubation solution (either PBSCM or PBSCM containing 5 µM of the hSERT inhibitor S-Citalopram or MJ1-53 for non-specific uptake) for 25 min at RT. After pre-incubation, 40 µl incubation solution (either PBSCM containing 12 increasing 5-HT concentrations (four technical replicates) or PBSCM containing 5-HT and inhibitor (technical duplicates)) was added to each well. Final 5-HT concentrations ranged from 0 to 10 µM for hSERT uptake assays and 0 to 1000 µM for hOCT2 uptake assays. A monoclonal cell line with low iSeroSnFR expression (70–80% of all cells showed sensor expression and around 20% of all cells showed high sensor expression) was used for all experiments. The specific uptake of 5-HT was determined by subtracting the mean non-specific uptake in the presence of either S-Citalopram or MJ1-53 from the total uptake and normalized to the maximal uptake capacity (V_max_). Data were fitted in Graphpad Prism to the in-built model for Michaelis–Menten kinetics using robust non-linear regression to calculate K_m_ and V_max_.

For the **uptake inhibition assay** determining IC_50_ values, the pre-incubation solutions contained 12 increasing concentrations of an hSERT or OCT2 inhibitor. HEK293MSR_iSeroSnFR cells used for the uptake inhibition assay were either monoclonal or polyclonal and showed varying expression levels of iSeroSnFR. The final 5-HT concentration used for the uptake inhibition assay, was 10 µM for hSERT and 500 µM for hOCT2. The change in fluorescence was determined 10 min after adding the incubation solution. For drugs with low potency, 20 µM MJ1-53 was used to constrain the bottom plateau. The raw data was fitted to a non-linear fit in GraphPad Prism ([inhibitor] vs response (three parameters) for hSERT data and [inhibitor] vs response – variable slope (four parameters) for hOCT2 data) and normalized to the obtained top and bottom plateau as follows: $\frac{raw data-bottom plateau}{top plateau-bottom plateau}$. The same non-linear fit of the normalized data was used to calculate logIC_50_ and IC_50_ values. IC_50_ values were converted to pK_i_ values using the Cheng-Prusoff equation^33^.

$${pK}_{i}= \frac{p{IC}_{50}}{1+ \frac{[S]}{{K}_{m}}}$$

For calculating pK_i_ values for hSERT, a K_m_ value of 1 µM was used based on previous studies in our lab, while for hOCT2 the K_m_ value determined in our assay (183.6 µM) was employed.

For the **efflux assay**, the cells were pre-incubated with PBSCM or PBSCM containing 10 µM 5-HT for 40 min, washed with PBSCM and incubated in 100 µl PBSCM or PBSCM containing an hSERT inhibitor for 5 min. Afterwards, 100 µl PBSCM containing a concentration range of an efflux inducing drug were added and the change in fluorescence was measured over time. The efflux inducing drug was added at time point 0 and a time window showing efflux in the linear range was chosen for further analysis. The fluorescence intensity measured in response to the drug was normalized to the mean fluorescence intensity measured 2.5 min, 5 min and 7.5 min before the addition of the drug. A simple linear regression was performed with GraphPad Prism for the time window showing efflux in the linear range and the slope of the linear decrease in fluorescence in response to the drug was plotted against increasing drug concentrations. A non-linear fit ([Inhibitor] vs. response (three parameters), GraphPad Prism) was used to calculate EC_50_ as well as the top and bottom plateau. The drug-induced efflux was calculated by subtracting the top plateau from the bottom plateau.

*Radiotracer assay* The radiotracer uptake inhibition assay was conducted two days post-transfection as described elsewhere^43^, except that 10 µM [^3^H]5-HT (10 µM, cold/hot ratio of 70) and HEK-293 MSR cells (Invitrogen) were used. Briefly, the cells were washed using a plate washer (Wellwash, Thermo Fisher Scientific), incubated in 40 µl pre-incubation solution (PBSCM or PBSCM containing 12 increasing concentrations of an hSERT inhibitor in duplicates) for 25 min at RT and incubated in 40 µl incubation solution (PBSCM, PBSCM containing radiolabeled 5-HT (10 µM, hot cold ratio of 70) or PBSCM containing both [^3^H]5-HT (10 µM, cold/hot ratio of 70) and a hSERT inhibitor) for 10 min at RT. The uptake was terminated by washing the cells with ice-cold PBSCM, 50 µl Microscint (Perkin Elmer) was added and radioactivity was counted in a TopCounter microplate scintillation counter approximately one hour after microscint addition. The raw data was fitted to a non-linear fit in GraphPad Prism ([inhibitor] vs response (three parameters)) and normalized to the obtained top and bottom plateau as follows: $\frac{raw data-bottom plateau}{top plateau-bottom plateau}$. The same non-linear fit of the normalized data was used to calculate logIC_50_ and IC_50_ values.

### Microscopy

*Fluorescence imaging* A Leica Stellaris 8 Confocal Microscope running the Leica Application Suite X software package equipped with an OPSL 488 nm laser (10.76% intensity), a HySD detector with an emission window of 498 to 650 nm running and the Leica Application Suite X software was used for imaging. Cells were washed three times prior to imaging and imaged in Hank’s Balanced Salt Solution (HBSS; Gibco) supplemented with 10 mM HEPES (pH = 7.4) using a 10x, air objective. Fluorescence was recorded at RT before and after the addition of 5-HT (final concentration: 10 µM) over a time course of 30 min with an image acquired every 30 s. Mock transfected cells and the hSERT inhibitor S-Citalopram (final concentration of 5 µM) were used as controls. For quantifying the fluorescence changes over time as a result of 5-HT uptake, images were analysed in ImageJ by using built-in threshold algorithms on the image stacks. Results were plotted using GraphPad 9 (Prism)^44^. Raw data was background corrected by dividing it with the fluorescence intensity obtained before adding 5-HT and normalized to the highest value obtained after 30 incubation with 5-HT. Representative images have been combined in ImageJ, the colour has been set to green and the brightness has been adjusted based on the image taken 10 min after 5-HT addition.

### Preprint

An earlier version of this manuscript was deposited as a preprint on Research Square, https://www.researchsquare.com/article/rs-3272320/v1..

### Supplementary Information

Supplementary Figures
