# Supplementary material for: 5-HT_FAsTR: a versatile, label-free, high-throughput, fluorescence-based microplate assay to quantify serotonin transport and release
Source: Sci Rep. 2024 Mar 19;14:6541. doi: 10.1038/s41598-024-56712-z (PMC10951269; doi:10.1038/s41598-024-56712-z)
Supplement: Supplementary file 1 — Supplementary Figures [file 41598_2024_56712_MOESM1_ESM.pdf]

Supplementary figures to "5-HT_FAsTR: A versatile, label-free, high-throughput microplate assay to quantify serotonin transport and release using the genetically encoded fluorescent biosensor iSeroSnFR"

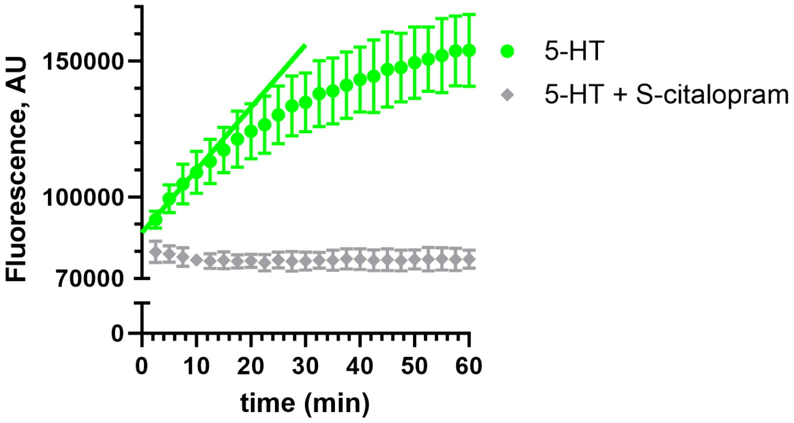

Figure S1: 5-HT uptake in 5HT_FAsTR does not saturate in assay conditions. HEK293MSR cells sstably transfected with iSeroSnFR were transiently transfected with hSERT in pCDNA3.1. Cells were subjected to a saturating concentration of 5-HT for SERT (6 µM) and the uptake of 5-HT was followed as development in fluorescence by repeated measurements in the same microplate wells. Green points show total uptake and grey points show non-specific uptake determined by preincubation and co-incubation with 5 µM of the SERT-selective inhibitor, S-citalopram. Shown is a representative experiment where points represent the mean of two technical replicates and error bars represent standard deviation. The line shows linear regression analysis of the points in the first 10 minutes of uptake of total uptake. Deviations from linearity is not evident within the first >20 minutes of the assay.

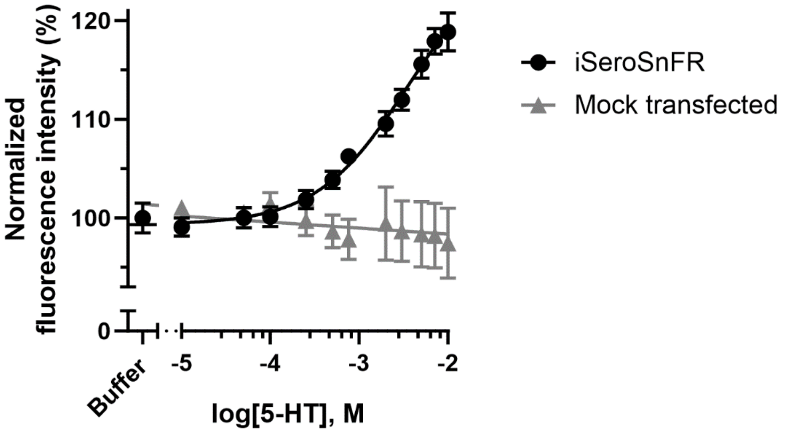

Figure S2: Dose-response curve of 5-HT binding to iSeroSnFR displays wide dynamic range of the fluorescent biosensor. HEK293MSR cells were transiently transfected with iSeroSnFR in the pMD vector using the extracellular form of the biosensor tethered to the membrane (Unger et al 2020) or empty pCDNA3.1 vector for mock transfection. Points represent mean normalized fluorescence values from three independent experiments. Shown is global data from three experimental replicate assays, each experiment with three technical replicates for iSeroSnFR and one technical replicate for mock transfected cells. Error bars represent SEM. Points were fitted to either a sigmoidal dose response curve (iSeroSnFR) or by linear regression (mock transfected). iSeroSnFR is not saturated at 10 mM 5-HT and shows an $EC_{50} \geq 1520$ µM.
